# Supplementary material for: A Transposon Story: From TE Content to TE Dynamic Invasion of Drosophila Genomes Using the Single-Molecule Sequencing Technology from Oxford Nanopore
Source: Cells. 2020 Jul 25;9(8):1776. doi: 10.3390/cells9081776 (PMC7465170; doi:10.3390/cells9081776)
Supplement: Supplementary file 1 [file cells-09-01776-s001.pdf]

## Isogenic wild type strains

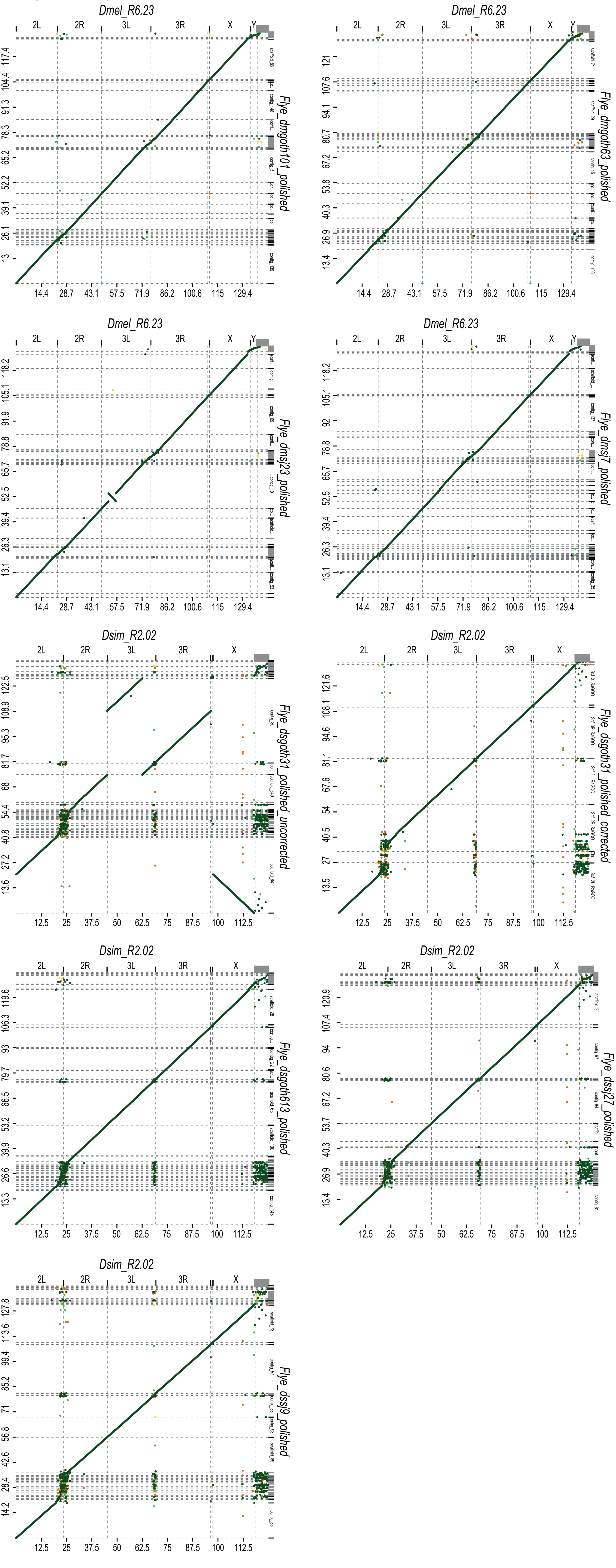


## Unstable strains

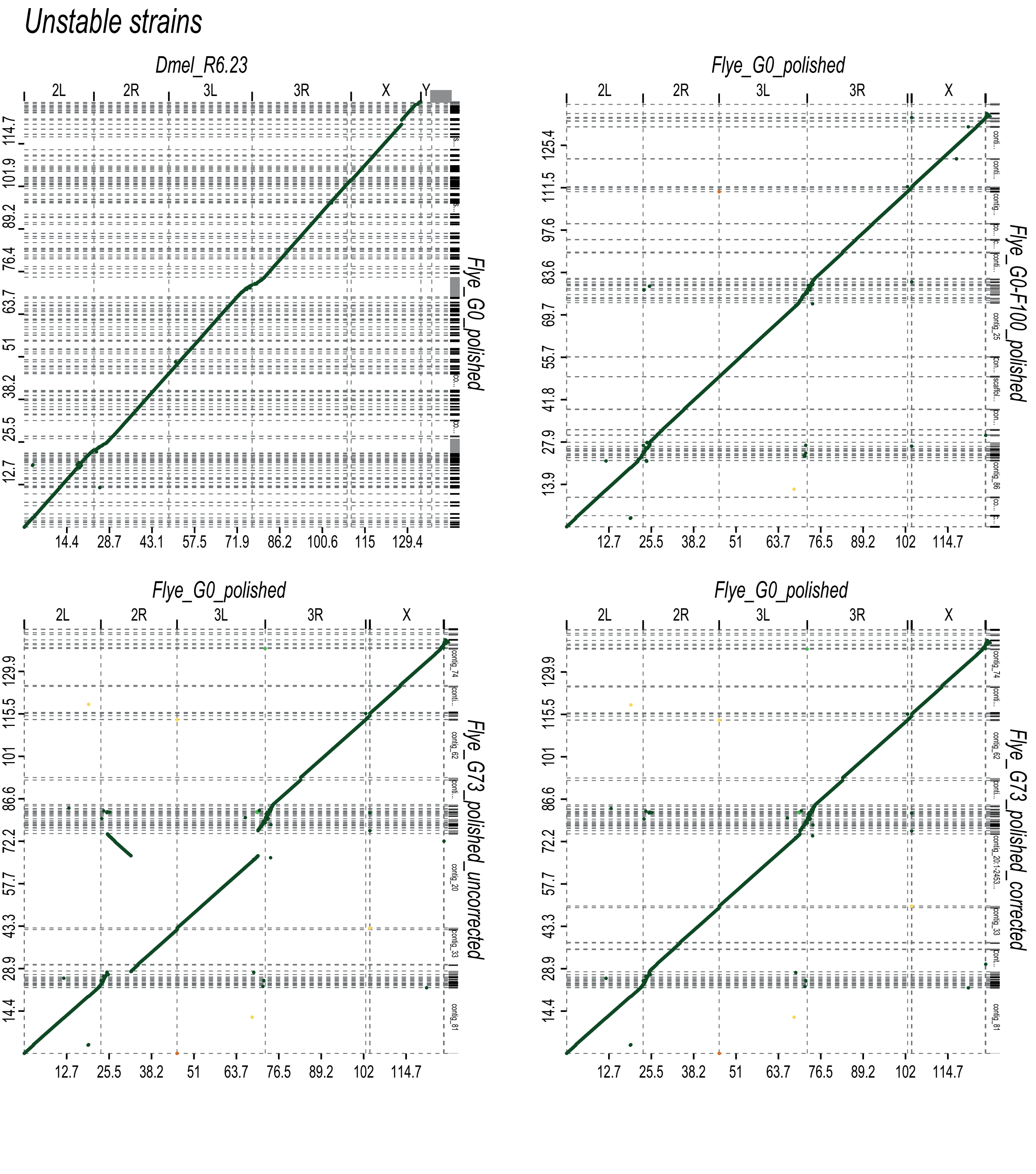


**Figure S1.** D-genies Genome-wide dot plot of ONT assembly contigs versus reference genome. On each subfigure the x-axis is the corresponding reference (Dsim or Dmel), with all chromosomes concatenated and shown. The y-axis represents the ONT assembly contigs, sorted based on their location on the reference genome. Each time a significant similarity is found, a dot is drawn. Multiple contiguous dots wil form a line. Scales on x- and y-axis are in million bp.

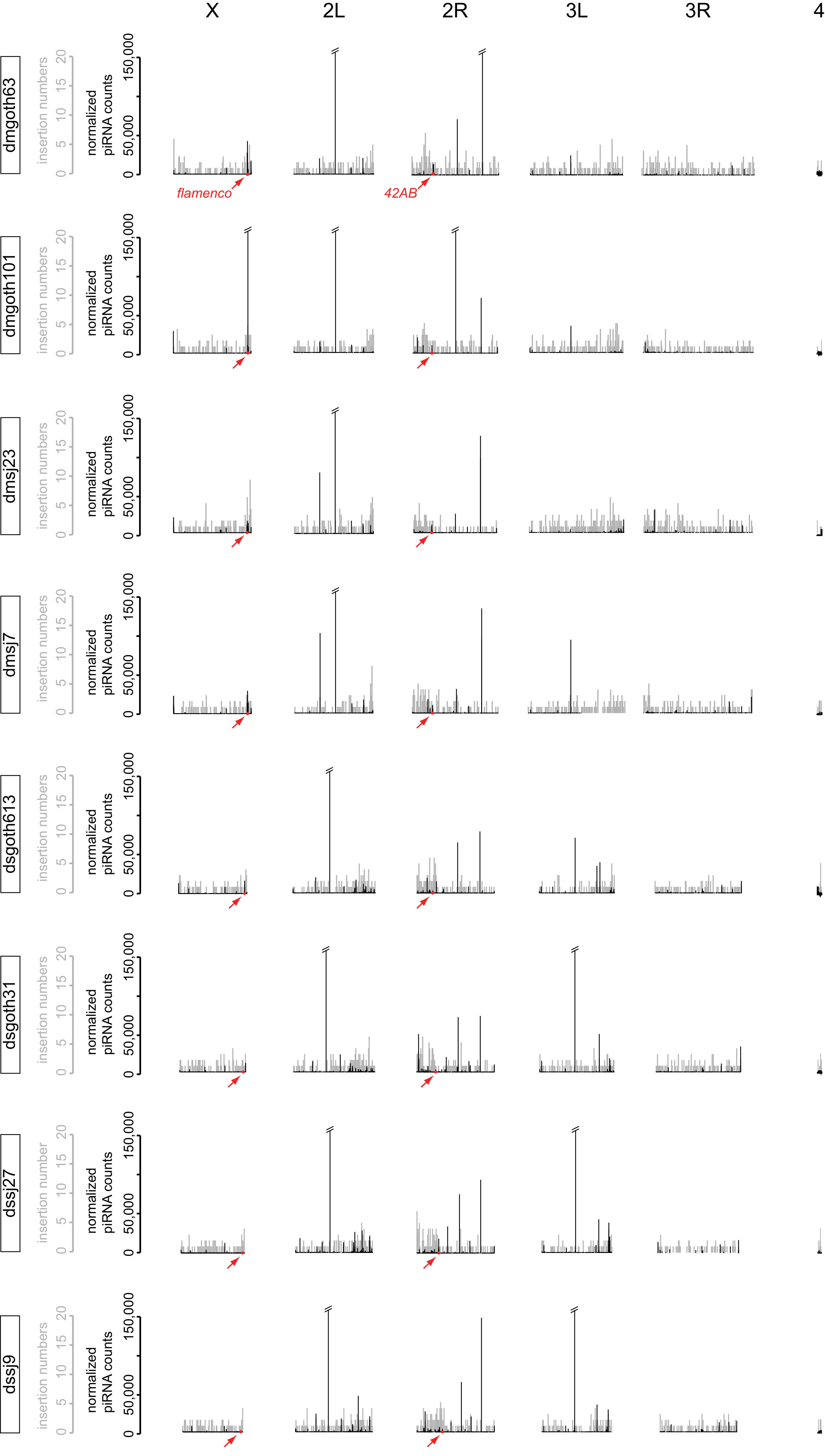


**Figure S2.** piRNA analyses in wild-type strains.Uniquely mapping piRNAs along ONT chromosome assemblies (black, normalized piRNA counts). Global variants identified along ONT chromosome assemblies (gray) for the isogenic wild type strains. Red arrows indicate flamenco (X chromosome) and 42AB (2R chromosome) piRNA clusters.

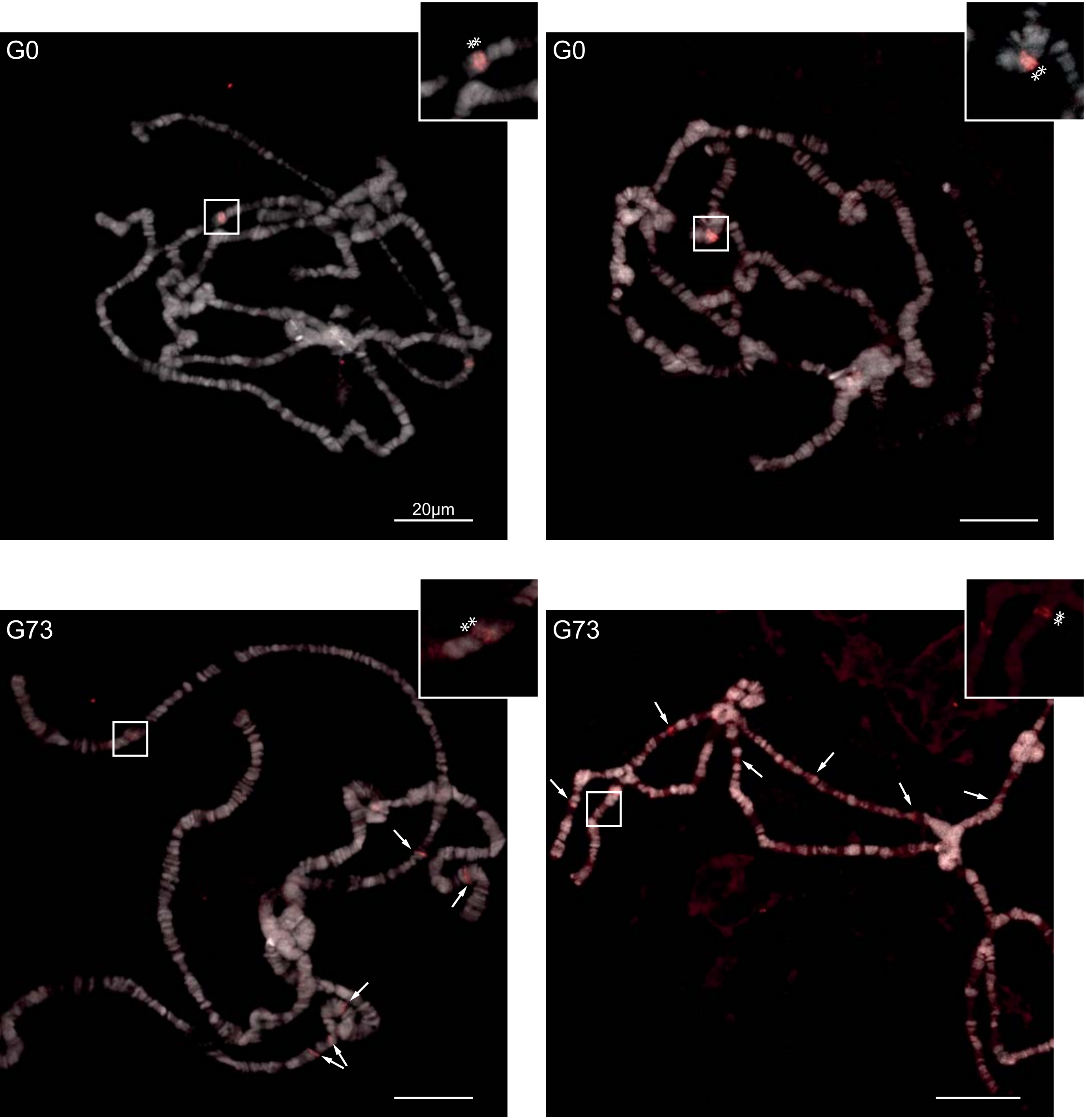


**Figure S3**. ZAM copies were visualized by fluorescent in situ hybridization on G0 (upper panels) and G73 (lower panels) polytene chromosomes. The two global variants corresponding to non-reference ZAM copies present in G0 and G73 were zoomed in and annotated by asterix. Arrows represent the ZAM new insertion in G73.

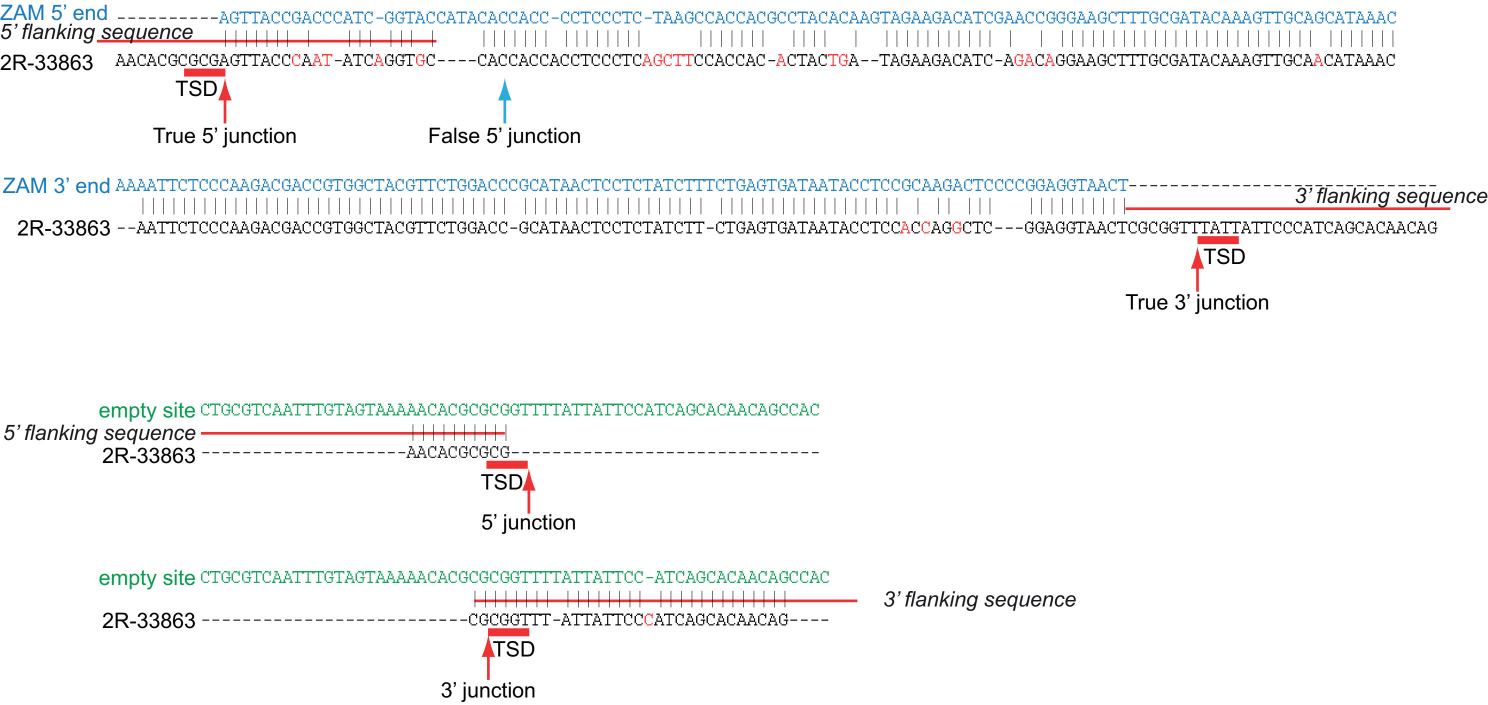


**Figure S4**. Alignments of the 2R-33863 insertion variant to the ZAM consensus sequence (blue lines) and to the empty site consensus sequence (green lines) are shown as examples of the proof reading procedure. The failure of BLAST to properly define the genome-5'LTR junction produced a 30 nts false 5' flanking sequence that did not contain the expected TSD at its very 3' end. Only the 10 nts true 5' flanking sequence matched to the empty site consensus sequence. The alignment of the 30 nts 3' flanking sequence with the empty site consensus sequence allowed to correct the sequencing errors of this single read ONT sequence.

**Table S1** : Statistics about sequencing data. All lengths are expressed in bases. Quality is expressed in standard Phred scale.

| Name | Number of Reads | N50 | Mean Qual, Phred | Total bases | Depth, in X |
|---|---|---|---|---|---|
| dmgoth101 | 4,947,537 | 6,005 | 11.2 | 19,024,837,157 | 149.8 |
| dmgoth63 | 4,246,836 | 9,812 | 9.6 | 22,619,307,622 | 174 |
| dmsj7 | 3,360,189 | 6,976 | 11.0 | 15,291,627,324 | 120.5 |
| dmsj23 | 3,587,974 | 7,282 | 10.8 | 16,490,294,931 | 129.8 |
| dsgoth31 | 3,583,002 | 8,666 | 11.0 | 19,172,220,131 | 151 |
| dsgoth613 | 3,321,983 | 9,106 | 10.9 | 18,716,931,514 | 147.4 |
| dssj9 | 3,429,538 | 10,99 | 11.1 | 23,677,429,979 | 186.4 |
| dssj27 | 3,028,593 | 10,13 | 11.1 | 19,556,705,249 | 154 |
| G0 | 2,252,087 | 3,768 | 9.1 | 5,418,397,754 | 40.5 |
| G0-F100 | 3,358,451 | 11,5 | 12.5 | 16,964,518,776 | 133.5 |
| G73 | 3,567,671 | 19,65 | 12.4 | 24,980,845,478 | 196.7 |

**Table S2** : Genome size estimations using different methods

| Strain | ONT assembly | findGSE | Flow cytometry |
|---|---|---|---|
| dmgoth101 | 130,483,042 | 135,030,062 | 161,078,651 |
| dmgoth63 | 134,481,426 | 133,349,116 | 163,472,918 |
| dmsj23 | 131,331,777 | 137,166,925 | 161,646,896 |
| dmsj7 | 131,360,683 | 147,133,883 | 162,154,282 |
| dsgoth31 | 135,039,133 | 128,975,006 | 144,341,424 |
| dsgoth613 | 132,908,190 | 129,095,835 | 141,793,085 |
| dssj27 | 134,309,820 | 132,157,931 | 144,020,805 |
| dssj9 | 134,093,082 | 132,052,451 | 141,561,506 |

**Table S3** : Comparison of TEI distributions across TE groups using chi-square tests

| **dmgoth63** | | | | |
|---|---|---|---|---|
| observed | | DNA | LINE | LTR |
| | ONT | 186 | 143 | 170 |
| | Illumina | 318 | 527 | 502 |
| expected | | DNA | LINE | LTR |
| | ONT | 136 | 181 | 182 |
| | Illumina | 368 | 489 | 490 |
| p-value | 9.598e-09 | | | |

| **dmgoth101** | | | | |
|---|---|---|---|---|
| observed | | DNA | LINE | LTR |
| | ONT | 198 | 127 | 116 |
| | Illumina | 327 | 552 | 541 |
| expected | | DNA | LINE | LTR |
| | ONT | 124 | 161 | 155 |
| | Illumina | 401 | 518 | 501 |
| p-value | 5.006e-18 | | | |

**dmsj23**

| | | DNA | LINE | LTR |
|---|---|---|---|---|
| observed | | DNA | LINE | LTR |
| | ONT | 193 | 178 | 176 |
| | Illumina | 336 | 568 | 544 |
| expected | | DNA | LINE | LTR |
| | ONT | 145 | 204 | 197 |
| | Illumina | 384 | 541 | 522 |
| p-value | 3.3962e-07 | | | |

**dmsj7**

| | | | | |
|---|---|---|---|---|
| observed | | DNA | LINE | LTR |
| | ONT | 170 | 166 | 116 |
| | Illumina | 239 | 455 | 349 |
| expected | | DNA | LINE | LTR |
| | ONT | 124 | 188 | 141 |
| | Illumina | 285 | 433 | 324 |
| p-value | 2.9600e-08 | | | |

**dsgoth613**

| | | | | |
|---|---|---|---|---|
| observed | | DNA | LINE | LTR |
| | ONT | 223 | 102 | 65 |
| | Illumina | 220 | 131 | 139 |
| expected | | DNA | LINE | LTR |
| | ONT | 196 | 103 | 90 |
| | Illumina | 247 | 130 | 114 |
| p-value | 6.2545e-05 | | | |

**dsgoth31**

| | | | | |
|---|---|---|---|---|
| observed | | DNA | LINE | LTR |
| | ONT | 223 | 127 | 113 |
| | Illumina | 273 | 144 | 169 |
| expected | | DNA | LINE | LTR |
| | ONT | 219 | 120 | 124 |
| | Illumina | 277 | 151 | 157 |
| p-value | 0.2412183 | | | |

**dssj27**

| | | | | |
|---|---|---|---|---|
| observed | | DNA | LINE | LTR |
| | ONT | 195 | 112 | 76 |
| | Illumina | 237 | 151 | 146 |
| expected | | DNA | LINE | LTR |
| | ONT | 180 | 110 | 93 |
| | Illumina | 251 | 153 | 129 |
| p-value | 0.02637001 | | | |

| dssj9 | | | | |
|---|---|---|---|---|
| observed | | DNA | LINE | LTR |
| | ONT | 198 | 146 | 109 |
| | Illumina | 185 | 103 | 118 |
| expected | | DNA | LINE | LTR |
| | ONT | 202 | 131 | 120 |
| | Illumina | 181 | 118 | 107 |
| p-value | 0.05874316 | | | |

**Table S4** : piRNA cluster coordinates based on flanking genes in *de novo* assembled genomes.

| Flanking genes | Cytological localisation | Chromosome | start in G0 | stop in G0 | size G0 | size G0-F100 | size G73 |
|---|---|---|---|---|---|---|---|
| *Pld − jing* | 42AB | 2R | 4,139,388 | 4,236,825 | 97,437 | 129,907 | 258,386 |
| *DIP1 − 178783* | 20A | X | 21,170,367 | 21,450,367 | 280,000 | 280,000 | 280,000 |
| *Kua − spir* | 38C | 2L | 20,192,754 | 20,355,333 | 162,579 | 169,704 | 162,334 |
| *scro − ND-AGGG* | CEN | 3L | 24,428,108 | 24,604,530 | 176,422 | 202,634 | 202,726 |
| *nrm − AGO3* | 80DEF | 3L | 22,930,499 | 23,378,676 | 448,177 | 460,709 | 460,740 |
| *AGO3 − nvd* | CEN | 3L | 2,3526,166 | 24,104,152 | 577,986 | 869,322 | 687,707 |
| *CG41099 − aux* | 81f | 3R | 2,085,834 | 2,364,369 | 278,535 | 500,287 | 765,130 |
| *Gprk1 − Ir41a* | 41C | 2R | 2,413,732 | 2,841,015 | 427,283 | 455,687 | 456,130 |
| *su(f) − 395000* | CEN | X | 22,021,754 | 22,136,858 | 115,104 | 131,972 | 270,635 |
| *onecut − unc-13* | 102DE | 4 | 562,380 | 793,565 | 231,185 | 272,369 | 274,590 |
| *fog − FucTC* | CEN | X | 21,781,855 | 21,917,690 | 135,835 | 148,861 | 141,887 |
| *Myo81F − Myo81F* | CEN | 3R | 4,872 | 19,81,126 | 1,976,254 | 1,979,008 | 1,979,679 |
| *CG17683 − Gprk1* | CEN | 2R | 2,121,549 | 2,283,262 | 161,713 | 192,541 | 193,065 |
| *Cht10 − CG12567* | CEN | 2L | 22,728,653 | 22,815,840 | 87,187 | 94,128 | 159,252 |
| *l(2)41ab − CG17691* | CEN | 2R | 689,228 | 757,459 | 68,231 | 248,084 | 267,191 |
| *unc-13 − CaMKII* | 102F | 4 | 845,580 | 964,531 | 118,951 | 128,666 | 128,578 |
| *Rpl5 − CG40006* | 40F | 2L | 22,331,808 | 22,582,287 | 250,479 | 81,406 | 81,436 |
| *MFS17 − CG41378* | CEN | 2R | 1,290,369 | 1,389,337 | 98,968 | 236,164 | 207,320 |
| *Cadps − 70000* | 102F | 4 | 11,92,128 | 1,208,671 | 16,543 | 63,830 | 20,093 |
| *Clamp − Marf1* | 40F | 2L | 22,083,442 | 22,210,653 | 127,211 | 131,134 | 131,137 |
| *cr45227 − Rya* | CEN | 2R | 1,840,746 | 1,915,110 | 74,364 | 175,004 | 164,380 |
| *DIP-λ − DIP-λ* | CEN | 2R | 10,903 | 383,636 | 372,733 | 389,529 | 391,836 |
| *sxc − ZnT41F* | 41F | 2R | 3,243,711 | 3,662,606 | 418,895 | 451,517 | 453,054 |
| *CG41378 − Scp1* | CEN | 2R | 1,467,508 | 1,558,280 | 90,772 | 81,823 | 81,812 |
| *lovit − FASN3* | CEN | 3L | 24,831,165 | 25,154,312 | 323,147 | 194,601 | 194,692 |
| *CG12567 − Tim23* | CEN | 2L | 22,833,265 | 22,895,924 | 62,659 | 132,984 | 133,277 |
| *CG9380 − 55000* | TEL | 2R | 22,858,949 | 22,874,018 | 15,069 | 55,000 | 55,000 |
| *His-ψ:cr33867 − eEF2* | 39E | 2L | 21,457,791 | 21,588,288 | 130,497 | 130,946 | 130,993 |
| *FASN3 − cr41320* | CEN | 3L | 23,397,946 | 25,168,256 | 1,770,310 | 400,583 | 689,251 |
| *eIF4B − 65000* | CEN | 3L | 26,372,541 | 26,437,541 | 65,000 | 65,000 | 65,000 |
| *zfh2 − Gat* | 102C | 4 | 497,061 | 580,471 | 83,410 | 85,021 | 85,222 |
| *kto − Su(z)12* | 76D | 3L | 19,778,945 | 19,837,552 | 58,607 | 584,92 | 58,503 |

| | | | | | | | |
|---|---|---|---|---|---|---|---|
| *DIP-λ − 55000* | CEN | 2R | 383,636 | 438,636 | 55,000 | 55000 | 55,000 |
| *cr40190 − CG40191* | CEN | 2R | 517,327 | 544,423 | 27,096 | 46,450 | 46,455 |
| *CG40006 − CG40006* | CEN | 2L | 2,258,2287 | 22,610,622 | 28,335 | 137,847 | 137,865 |
| *RanGAP − CG10194* | 37E | 2L | 19515,721 | 19,564,447 | 48,726 | 48,652 | 48,661 |
| *rdgA − rdgA* | 8C | X | 8,590,101 | 8,709,328 | 119,227 | 118,760 | 118,612 |
| *CG33552 − VhaSFD* | 36A | 2L | 16,548,760 | 16,584,034 | 35,274 | 35,169 | 35,171 |
| *CG8407 − RpS11* | 48E | 2R | 9,844,285 | 9,885,097 | 40,812 | 40,779 | 40,784 |
| *Smr − CG32647* | 11B | X | 12,412,625 | 12,440,875 | 28,250 | 28,790 | 28,822 |
| *Dbp80 − Dbp80* | CEN | 3L | 26,056,652 | 262,12,254 | 155,602 | 146,817 | 155,815 |
| *Cyt-c1L − cg11951* | 99A | 3R | 27,328,861 | 27,356,079 | 27,218 | 27,165 | 27,171 |
